# Eco-Friendly g-C_3_N_4_/Carboxymethyl Cellulose/Alginate Composite Hydrogels for Simultaneous Photocatalytic Degradation of Organic Dye Pollutants

**DOI:** 10.3390/ijms25147896

**Published:** 2024-07-19

**Authors:** Ksenija Milošević, Davor Lončarević, Melina Kalagasidis Krušić, Milica Hadnađev-Kostić, Jasmina Dostanić

**Affiliations:** 1 Department of Catalysis and Chemical Engineering, Institute of Chemistry, Technology and Metallurgy, National Institute of Republic of Serbia, University of Belgrade, Njegoševa 12, 11000 Belgrade, Serbia; dloncarevic@nanosys.ihtm.bg.ac.rs (D.L.); jasmina.dostanic@ihtm.bg.ac.rs (J.D.); 2 Faculty of Technology and Metallurgy, University of Belgrade, Karnegijeva 4, 11000 Belgrade, Serbia; meli@tmf.bg.ac.rs; 3 Faculty of Technology Novi Sad, University of Novi Sad, Bulevar Cara Lazara 1, 21102 Novi Sad, Serbia; hadnadjev@tf.uns.ac.rs

**Keywords:** graphitic carbon nitride, composite hydrogels, eco-friendly material, photocatalysis, wastewater remediation

## Abstract

The presented study was focused on the simple, eco-friendly synthesis of composite hydrogels of crosslinked carboxymethyl cellulose (CMC)/alginate (SA) with encapsulated g-C_3_N_4_ nanoparticles. The structural, textural, morphological, optical, and mechanical properties were determined using different methods. The encapsulation of g-C_3_N_4_ into CMC/SA copolymer resulted in the formation of composite hydrogels with a coherent structure, enhanced porosity, excellent photostability, and good adhesion. The ability of composite hydrogels to eliminate structurally different dyes with the same or opposite charge properties (cationic Methylene Blue and anionic Orange G and Remazol Brilliant Blue R) in both single- and binary-dye systems was examined through adsorption and photocatalytic reactions. The interactions between the dyes and g-C_3_N_4_ and the negatively charged CMC/SA copolymers had a notable influence on both the adsorption capacity and photodegradation efficiency of the prepared composites. Scavenger studies and leaching tests were conducted to gain insights into the primary reactive species and to assess the stability and long-term performance of the g-C_3_N_4_/CMC/SA beads. The commendable photocatalytic activity and excellent recyclability, coupled with the elimination of costly catalyst separation requirements, render the g-C_3_N_4_/CMC/SA composite hydrogels cost-effective and environmentally friendly materials, and strongly support their selection for tackling environmental pollution issues.

## 1. Introduction

The widespread industrial pollution from the negligent continuous discharge of harmful materials into clean water bodies contaminates both surface and groundwater, causing significant environmental damage. These harmful substances, such as organic dyes, are usually non-biodegradable, highly toxic, thermally stable, photo-resistant, and often intensely colored, posing threats to both human health and the environment [1,2]. Consequently, wastewater remediation and the protection of clean water bodies are urgent global concerns and present a challenging task for scientists worldwide. Numerous purification methods have been proposed, including ion-exchange removal, adsorption, membrane separation techniques, ozonation, photocatalysis, and coagulation [3,4]. Among the mentioned methods, heterogeneous photocatalysis stands out due to its effectiveness, economic feasibility, and environmental friendliness, using solar energy to completely degrade pollutants without generating secondary waste. Recent research is focused on the development of a promising photocatalyst with an optimal combination of electronic structure and light absorption properties, as well as with the efficient separation of photogenerated charge carriers, such as the semiconductors TiO_2_, r-GO, g-C_3_N_4_, and ZnO [5,6,7,8]. Graphitic carbon nitride (g-C_3_N_4_) has garnered attention due to its metal-free structure, element abundance in nature, facile preparation, enhanced visible light harvesting, high thermal and chemical stability, excellent electronic structure, and cost-effectiveness, making it an environmentally sustainable photocatalyst [9,10]. However, the problems associated with the high recombination rate of photogenerated charge carriers, low photocatalytic activity, substantial agglomeration, and difficult separation from the reaction solution may restrict the large-scale application and cyclic reusability [11,12]. Relevant studies have shown that the immobilization of photocatalysts on different supports improves the photocatalytic performance, prevents or reduces agglomeration, and provides stable active sites for the reaction to occur, which is suitable for large-scale application [13]. So far, different supports, both inorganic and organic, have been successfully applied for the immobilization of photocatalysts [14,15,16,17,18]. The difficulties associated with the high cost and poor adherence between photocatalysts and inorganic supports have moved the interest toward polymer supports.

In recent years, numerous semiconductors have been immobilized on various synthetic polymers, such as polyethylene, polypropylene, polystyrene, poly(vinyl chloride), etc., and successfully applied in wastewater remediation [19,20,21,22]. Although synthetic polymers have been effectively used as supports, their high cost, limited availability, and environmental concerns emphasize the need for environmentally friendly and cost-effective natural polymers. Thomas et al. [21] developed a novel hydrogel by encapsulating TiO_2_ nanoparticles in barium ion-crosslinked alginate/carboxymethyl cellulose for the photocatalytic degradation of Congo Red dye. Consequently, different polysaccharides have gained attention due to their sustainability, non-toxicity, biodegradability, and environmentally friendliness coupled with their excellent selectivity, biocompatibility, adsorption capacity, and recyclability [23,24,25]. Polysaccharides form hydrogels, an important class of polymeric materials known for their swelling properties, soft texture, and shape persistence. Hydrogels facilitate the easy diffusion of molecules into the gel network, making polysaccharides suitable for various applications [26,27,28,29,30]. There are only a few studies that focus on the immobilization of g-C_3_N_4_ on polysaccharides, hydrogels, and hydrogel-based composites for photocatalytic reactions. Zhao et al. [31] developed a stable and reusable g-C_3_N_4_/sodium alginate self-cleaning membrane, achieving the excellent dye removal of different cationic and anionic dyes. For photocatalytic NO removal, Li et al. [32] used the carboxymethyl tamarind kernel polysaccharide as a support for Au-doped g-C_3_N_4_ nanosheets, which showed an excellent efficiency.

Carboxymethyl cellulose (CMC) and sodium alginate (SA) are widely applied materials due to their high stability, biocompatibility, cost-effectiveness, and the presence of hydroxyl and carboxyl functional groups [33,34]. Pristine CMC cannot maintain a stable structure in an aqueous environment, has a low adsorption efficiency, and requires modification in order to improve its stability [35]. Even though it is a common practice to crosslink SA with divalent and multivalent cations, SA hydrogels have been shown to be very brittle [36]. The improvement of SA and CMC hydrogels through polymer mixing, dual crosslinking, and filler agent addition [37,38] results in hydrogels with not only enhanced mechanical properties but also improved thermal stability, chemical resistance, and swelling capacity. The utilization of graphitic carbon nitride (g-C_3_N_4_) offers a promising approach not only to enhance the performance of hydrogels for environmental remediation but also to enable better synergistic effects of the adsorption and photodegradation of organic pollutants, beyond merely improving hydrogel properties [39,40,41].

In light of the specific requirements for improved properties, the primary objective of this study was to develop eco-friendly, photostable, and biodegradable composite hydrogel beads based on CMC, SA, and g-C_3_N_4_ through a simple synthesis using the environmentally friendly crosslinkers CaCl_2_ and citric acid. As an essential part of the study, the photocatalytic activity of the as-synthesized beads in the degradation of anionic and cationic dyes, in single and simultaneous systems, was investigated. Moreover, the investigation into the radical scavenger study of the photocatalytic degradation and the assessment of the photocatalytic activity in multiple cycles will provide valuable insights into the photocatalytic mechanism, offering significant contributions to the field.

## 2. Results and Discussion

### 2.1. Composite Hydrogel Characterization

**Figure 1:**
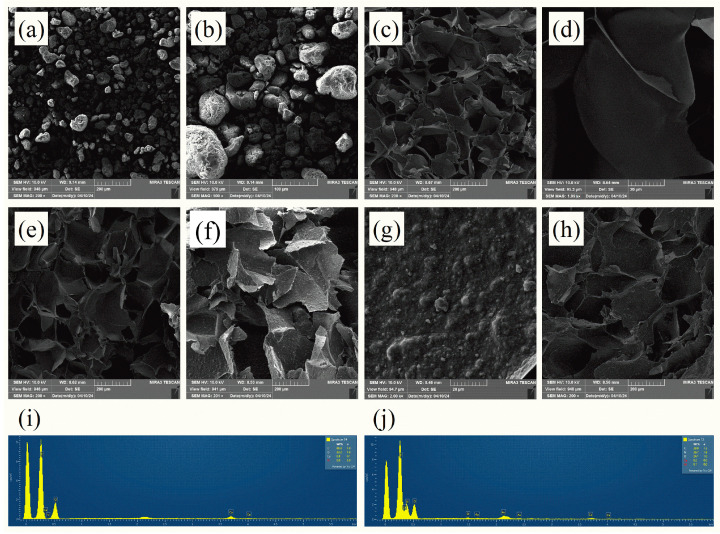
FE-SEM images of the (**a**,**b**) pristine g-C_3_N_4_, the pristine polymer hydrogels (**c**,**d**) CMC/SA_1:1_ and (**e**) CMC/SA_2:1_, and the composite hydrogels (**f**,**g**) g-C_3_N_4_/CMC/SA_1:1_ and (**h**) g-C_3_N_4_/CMC/SA_2:1_ at different magnifications; the EDX spectra of (**i**) CMC/SA_2:1_ and (**j**) g-C_3_N_4_/CMC/SA_2:1_.

The surface morphologies of the pristine g-C_3_N_4_, polymer hydrogels (CMC/SA), and composite hydrogels (g-C_3_N_4_/CMC/SA beads) were characterized by Field Emission Scanning Electron Microscopy (FE-SEM), as shown in Figure 1a–h. The FE-SEM micrographs of the g-C_3_N_4_ revealed the structure of porous nanoparticles with irregular shapes. The cross-section of the CMC/SA beads showed regular sheet-like morphologies with smooth surfaces. The g-C_3_N_4_/CMC/SA micrographs also displayed regular sheet-like morphologies with the uneven or rugged-like structure of g-C_3_N_4_ nanoparticles uniformly dispersed into the polymer matrix. The CMC/SA and g-C_3_N_4_/CMC/SA micrographs illustrated that all of the hydrogels possessed homogeneous surfaces. From the micrographs, it was observed that there was no significant morphological difference between the hydrogels with different polymer ratios of CMC/SA_1:1_ compared to CMC/SA_2:1_ and g-C_3_N_4_/CMC/SA_1:1_ to g-C_3_N_4_/CMC/SA_2:1_.

The energy-dispersive X-ray (EDX) spectra of the polymer hydrogels (CMC/SA_2:1_) and composite hydrogels (g-C_3_N_4_/CMC/SA_2:1_) are shown in Figure 1i,j. The EDX spectra of the samples confirmed the presence of C, O, and Ca elements in the structures of both hydrogel samples. Additionally, the detection of N in the g-C_3_N_4_/CMC/SA_2:1_ sample verified the successful encapsulation of g-C_3_N_4_ into the polymer matrix.

**Figure 2:**
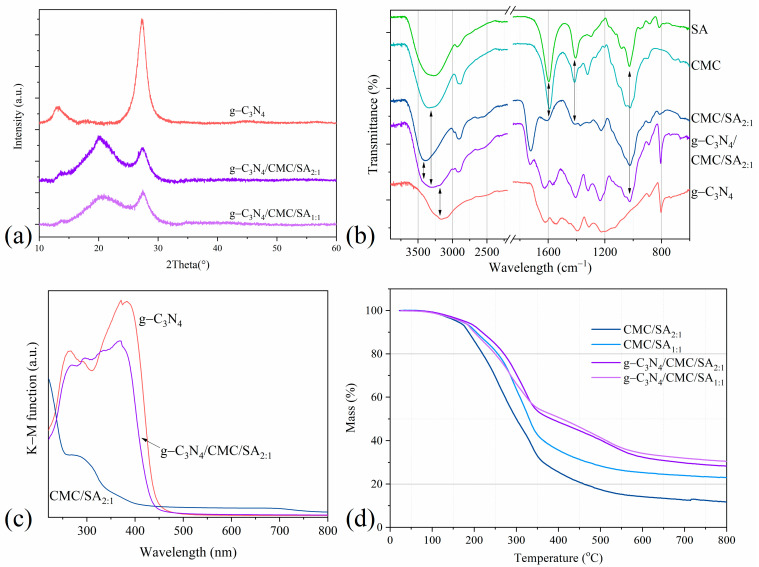
(**a**) XRD patterns, (**b**) ATR-FTIR spectra, (**c**) normalized diffuse reflectance spectra (Kubelka–Munk function), and (**d**) TGA thermographs of pristine g-C_3_N_4_, pristine polymer hydrogels, and nanocomposite hydrogels.

The X-ray diffraction (XRD) patterns of the pristine g-C_3_N_4_ and g-C_3_N_4_/CMC/SA beads are presented in Figure 2a. The XRD pattern of g-C_3_N_4_ exhibited two characteristic peaks at 13.1° and 27.2°, which corresponded to the (001) and (002) crystal planes. The peak at 13.1° could be assigned to tris-triazine repeating units, whereas the most intense peak at 27.2° was associated with the interplanar stacked graphitic hexagonal layered structure (PDF#87-1526) [42]. The high-intensity broad diffraction peak at 20.1° was designated to the CMC/SA structure [43], whereas the peak broadness indicated the amorphous structure of the polymers.

The Attenuated Total Reflectance–Fourier Transform Infrared (ATR-FTIR) spectra of the pristine precursors, polymer hydrogels, and composite hydrogels are given in Figure 2b. The ATR-FTIR spectra of the CMC and SA showed bands at 1590 cm^−1^ and 1410 cm^−1^, which can be attributed to the asymmetric and symmetric stretching vibrations of the carboxylate anion [39,44]. Two additional characteristic infrared stretching absorptions, broad at 3300 cm^−1^ and intense at 1020 cm^−1^, were observed and are attributed to O-H and C-O bonds [31]. Additionally, the ATR-FTIR spectra of the formed CMC/SA beads revealed the presence of a carbonyl band at 1726 cm^−1^ [45] and a slight band shift at 3300 cm^−1^. The spectra of the pristine g-C_3_N_4_ revealed typical graphitic carbon nitride bands assigned to C=N and C-N stretches in the regions of 1650–1200 cm^−1^ and to the breathing mode of tri-s-triazine units at 806 cm^−1^ [39,46]. The additional broad band at around 3160 cm^−1^ was indicative of the terminal amino group N–H stretching vibration [46]. After the encapsulation of g-C_3_N_4_ in the CMC/SA matrix (Figure 2b), absorption bands at around 3160 cm^−1^ and 3400 cm^−1^ merged and broadened, while the blue shift of the bands in the 1650–1200 cm^−1^ region indicated the interaction between the g-C_3_N_4_ and CMC/SA matrix.

The results from the diffuse reflectance measurements (DRSs) presented in Figure 2c indicate negligible band-gap modulations upon g-C_3_N_4_ encapsulation into the polymer matrix in comparison with the band gap of the pristine g-C_3_N_4_. The energy band gap of the pristine g-C_3_N_4_ was found to be 2.88 eV, which is in agreement with the reported literature data [9]. The optical band gap of g-C_3_N_4_/CMC/SA_2:1_ was observed to be 2.98 eV. The band gap calculation for the pristine CMC/SA_2:1_ hydrogels was challenging considering their non-conductive nature.

Considering that the thermal stability of polymers is one of the crucial parameters for high-temperature applications, Thermogravimetric analysis (TGA) was conducted on the prepared polymer and composite hydrogels. The TGA thermograms of the CMC/SA and g-C_3_N_4_/CMC/SA beads are presented in Figure 2d. The three main regions of weight loss could be distinguished in all of the investigated samples. The first weight loss detected in the range from 80 °C to 180 °C was attributed to the evaporation of free water from the polymer network. In the range from 180 °C to 330 °C, the second weight loss was observed and ascribed to the thermal degradation of unstable oxygen-containing functional groups, namely, hydroxyl and carboxyl groups, and glycosidic bonds. Furthermore, the third weight loss was distinguished in the range from 350 °C to 550 °C and could be associated with the thermal destruction of polymer molecular chains. The TGA curves revealed that the percentage of the weight loss of CMC/SA_1:1_, CMC/SA_2:1_, g-C_3_N_4_/CMC/SA_1:1_, and g-C_3_N_4_/CMC/SA_2:1_ at 800 °C was 77%, 88%, 70%, and 72%, respectively. To facilitate further comparison, the temperature corresponding to 5% and 30% weight loss of the initial mass, together with the heat-resistance index, are presented (Table 1). From the table, it can be observed that there is a slight difference in temperatures corresponding to a 5% weight loss between the g-C_3_N_4_/CMC/SA_1:1_ composite and its polymer counterpart. However, a more pronounced difference in T_5%_ was obtained between the g-C_3_N_4_/CMC/SA_2:1_ and CMC/SA_2:1_ samples. Comparing the temperatures for the 50% conversion revealed the lower weight loss of both composite hydrogels compared to that of the pristine polymer hydrogels, indicating that the thermal stability of the composite hydrogels was enhanced by g-C_3_N_4_ encapsulation. Further estimation of the thermal stability could be confirmed by the heat-resistance index (T_HRI_) of the samples, which provides information on the ability of a material to resist a heat flow (Table 1). From the given data, the highest heat-resistance index was observed for g-C_3_N_4_/CMC/SA_2:1_, followed by g-C_3_N_4_/CMC/SA_1:1_, indicating the better pyrolytic resistance of the composite hydrogels compared to pristine polymer hydrogels.

To explore the mechanical properties of the pristine polymer and composite hydrogels, a series of tests were conducted. The corresponding values of the maximal compressive stress and maximal stroke strain are listed in Table 2. The results reveal that the mechanical properties of the pristine polymer hydrogels were strongly dependent on the CMC/SA ratio. The hydrogels with a higher percentage of CMC showed higher maximal compressive stress but with a lower maximal stroke strain. The maximal compressive stress of CMC/SA_1:1_ remained unchanged by g-C_3_N_4_ encapsulation. However, the maximal stroke strain of the CMC/SA_1:1_ beads was slightly enhanced by g-C_3_N_4_ encapsulation, indicating an increase in the flexibility of the composite hydrogels. On the other hand, the maximal compressive stress of CMC/SA_2:1_ was significantly reduced by g-C_3_N_4_ encapsulation, while the maximal stroke strain was enhanced. The maximal compressive stress was obtained for CMC/SA_2:1_, while the g-C_3_N_4_/CMC/SA_1:1_ sample exhibited the highest maximal stroke strain.

**Figure 3:**
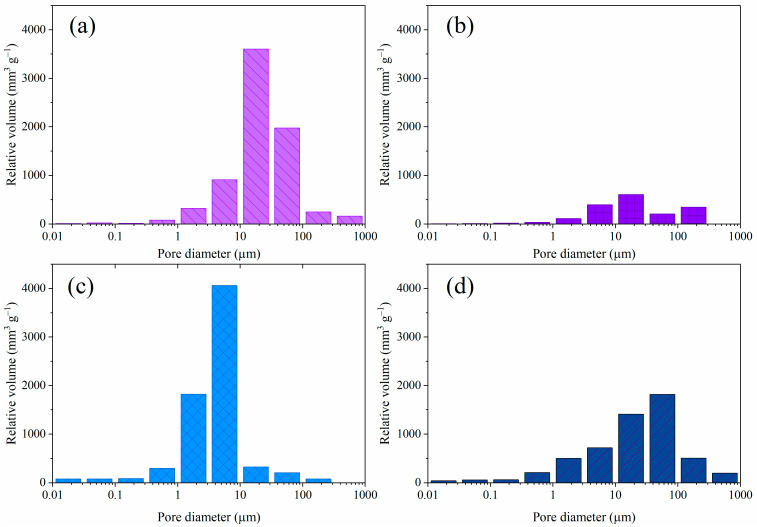
Pore size distribution of the pristine polymer hydrogels (**a**) CMC/SA_1:1_ and (**b**) CMC/SA_2:1_, and the composite hydrogels (**c**) g-C_3_N_4_/CMC/SA_1:1_ and (**d**) g-C_3_N_4_/CMC/SA_2:1_.

The pore size distribution histograms for CMC/SA_1:1_, CMC/SA_2:1_, g-C_3_N_4_/CMC/SA_1:1_, and g-C_3_N_4_/CMC/SA_2:1_ are displayed in Figure 3, while the total intruded specific volume and median pore diameter values are summarized in Table 3. The CMC/SA_1:1_ sample exhibited the highest total intruded specific volume, indicating the greatest porosity among the samples. Additionally, similar median pore diameters were observed for CMC/SA_1:1_ and CMC/SA_2:1_, despite differences in their total intruded specific volume.

The encapsulation of g-C_3_N_4_ into the CMC/SA_1:1_ polymer matrix resulted in the reduction in the median pore diameter, being significantly smaller compared to the other samples. Additionally, it should be noted that, during the Mercury Intrusion Porosimetry (MIP) measurements, the destruction of the spherical shape was observed for the CMC/SA_2:1_ sample, suggesting that there was a mechanical collapse of the beads. Consequently, the results for the g-C_3_N_4_/CMC/SA_2:1_ sample suggested that the encapsulation of g-C_3_N_4_ strengthened the structure of the hydrogel beads and achieved the expected specific pore volume. Moreover, the g-C_3_N_4_/CMC/SA_2:1_ exerted an increased specific pore volume and a similar median pore diameter compared to the pristine CMC/SA_2:1_. The pore diameter was determined through the Washburn equation from the applied pressure, directly indicating the inlet pore diameter. The intrusion and extrusion curves (Figure S1 in Supplementary Materials) revealed the presence of intrusion and the absence of extrusion, related directly to an ink-bottle pore type [47]. The ink-bottle pore type is characteristic for samples where narrow pore throats with small median pore diameters lead to smaller apparent pore diameters than their actual sizes [47]. The MIP measurements coupled with the SEM analysis could strongly elucidate the texture of the bead framework, suggesting the presence of large pores inside of the beads and smaller throat-like pores at the shells of the beads. 

### 2.2. Photocatalytic Activity of g-C_3_N_4_/CMC/SA_2:1_ Beads

#### 2.2.1. Single-Dye Photocatalytic Systems

The photocatalytic performance of the prepared pristine g-C_3_N_4_ and g-C_3_N_4_/CMC/SA_2:1_ beads was evaluated through the photodegradation of cationic Methylene Blue (MB) and anionic Orange G (OG) dyes in single-dye photocatalytic systems under simulated solar light irradiation. In order to rule out the possible direct photolysis of the dyes, preliminary tests were performed on both dyes without a photocatalyst, and it was observed that both dyes were photochemically stable and the photocatalytic reaction was solely responsible for the dye degradation.

**Figure 4:**
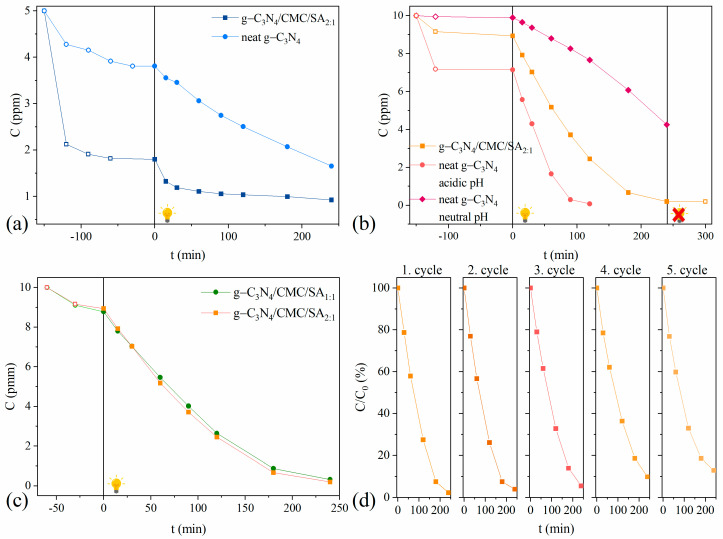
Photodegradation of (**a**) MB dye and (**b**) OG dye using pristine g-C_3_N_4_ and g-C_3_N_4_/CMC/SA_2:1_ beads; (**c**) OG dye using g-C_3_N_4_/CMC/SA_1:1_ and g-C_3_N_4_/CMC/SA_2:1_ beads; (**d**) OG dye using g-C_3_N_4_/CMC/SA_2:1_ beads in 5 consecutive cycles. [Experimental conditions: c_0_ (OG) = 10 ppm, c_0_ (MB) = 5 ppm, pH = 3.8, T = 25 °C, simulated solar light irradiation].

The time-dependent decolorization of the MB and OG dyes in single solutions is presented in Figure 4a,b. Prior to the photocatalytic degradation experiments, both suspensions were stirred under a dark atmosphere for 120 min to obtain adsorption/desorption equilibrium. Subsequently, the reaction mixture was irradiated under simulated sunlight for 4 h. After only 30 min of dark exposure, around 75% of the dye was adsorbed on composite hydrogel, indicating the high adsorption capacity of the MB dye onto the g-C_3_N_4_/CMC/SA_2:1_ beads (Figure 4a). From the observed results, the MB dye adsorption mechanism could involve physi- or chemisorption, which include van der Waals forces, intermolecular hydrogen bonding, π-π interactions, or hydrophobic interactions. MB is a cationic dye, and the zeta potentials of CMC and SA are known to be negative [48]. Therefore, it is expected that the electrostatic attraction between the negatively charged surface of the CMC/SA copolymer and positively charged cationic dye played an important role in the high MB adsorption on the composite surface. Since, in acidic media, the aliphatic N-H groups in g-C_3_N_4_ are being protonated, the MB dye was predominantly adsorbed on the polymer support.

After 4 h of irradiation, only 17.5% of the initial MB dye amount was degraded (Figure 4a), indicating that the high adsorption of the MB dye on the composite hydrogel consequently led to slow MB degradation. Increasing the amount of adsorbed dye on the surface of the composite hydrogel reduced the number of photons available to reach the catalyst surface. This led to the formation of fewer reactive species and subsequently decreased the degradation efficiency. Furthermore, the MB dye mainly adsorbed on the polymer, prevented the penetration of light, and reduced the photocatalytic activity. Similar results were reported by Wang et al., where photocatalysts supported by CMC showed excellent MB sorption capacity and the lower photodegradation of MB dye in comparison with photocatalysts without CMC [49].

To test the effect of encapsulation on the degradation performance, the adsorption and photocatalytic behavior of g-C_3_N_4_/CMC/SA_2:1_ beads was compared to pristine g-C_3_N_4_ (Figure 4a). A significantly lower adsorption of the MB dye on g-C_3_N_4_ was observed compared to the composite hydrogel due to the reduced electrostatic interaction between the cationic MB dye molecule and the protonated g-C_3_N_4_ surface. On the contrary, g-C_3_N_4_ demonstrated slightly higher photocatalytic activity in MB degradation compared to the composite hydrogel. This is probably due to the less pronounced external diffusion limitations, which enhanced the accessibility of the g-C_3_N_4_ catalytic surface to photons and reactant molecules compared to the composite hydrogel.

The adsorption and photocatalytic performance of the pristine g-C_3_N_4_ and g-C_3_N_4_/CMC/SA_2:1_ beads were also evaluated utilizing OG dye as a model anionic dye. The results revealed the lower adsorption capacity and sluggish adsorption kinetics of the OG dye onto the composite hydrogel compared to g-C_3_N_4_ (Figure 4b). Considering that both CMC and SA polysaccharides are rich in HO- and COO- functional groups, these groups were responsible for the electrostatic repulsion of the anionic OG dye. On the other hand, the aliphatic N-H groups present in g-C_3_N_4_ are partially protonated under acidic conditions, which additionally contributed to the reduction in the electrostatic repulsion and the higher adsorption of the OG dye onto the g-C_3_N_4_ surface. Furthermore, a significantly higher photodegradation rate of the OG dye compared to the MB dye was observed, since 99% of the OG dye was degraded within 4 h of light irradiation. The repulsive forces between the anionic dye molecules and the polymer matrix helped in preventing dye aggregation within the hydrogel. This dispersion of dye molecules facilitated their accessibility to the surrounding solvent and enhanced the overall efficiency of the dye removal process.

The distinct reactivity of MB and OG dyes arises from their significantly different chemical structures, which, in turn, leads to different degradation pathways during the photocatalytic degradation process. The MB dye molecule has a phenothiazine structure, which consists of two benzene rings attached to a central thiazine ring with two dimethyl amino groups, and it is more susceptible to nucleophilic attack or oxidation at specific sites [50]. On the other hand, OG dye belongs to azo dyes, and degradation usually includes the cleavage of the azo group or other types of reactions involving the electrophilic attack on the azo functionality [51]. Depending on the generated reaction species, varying dye structures, sizes, and degradation mechanisms, different intermediates may form, affecting the rates of adsorption and photodegradation.

An overview of various photocatalytic materials with the pollutant concentration and removal efficiency is provided in Table 4. However, one must be aware that the direct comparison of the results is challenging due to the differences in experimental set-ups across studies. Heidarpour et al. [52] reported that photoactive Ag/AgCl encapsulated into CMC with different crosslinking agents, AlCl_3_ or FeCl_3_, showed a progressive increase in the photodegradation of Rhodamine B dye. The TiO_2_ immobilized into Fe^3+^-crosslinked ternary CMC/polyaniline exhibited the complete photodegradation of Cristal Violet dye [53]. The photocatalyst encapsulated into hydrogel in spherical form was fabricated by Hao et al. [39] when g-C_3_N_4_/calcium alginate hydrogels were produced and employed for the photocatalytic degradation of anionic and cationic dyes. Additionally, the excellent photocatalytic activity of nitrogen-doped graphene oxide/ZnO/ZnO_2_ encapsulated into CMC was reported by Xue et al. [54]. In comparison to similar studies conducted in recent years, the results presented in our study demonstrate that the utilization of g-C_3_N_4_ encapsulated into the CMC/SA polymer matrix yields numerous advantages in the realm of photocatalysis while staying up-to-date with current developments.

Suspended “slurry-type” systems using powdered catalysts are the most widely applied systems, typically exhibiting high photocatalytic efficiency, due to the absence of mass transfer limitations. However, these systems face challenges with recovery and separation. The encapsulation strategy addresses these issues, but usually reduces the accessibility of the catalytic surface (reactive surface area) to the photons and the reactants due to external diffusion limitations, leading to lower catalytic performance. The results from our study showed that the encapsulation of g-C_3_N_4_ into the polymer matrix results in a slight, but not significant, reduction in the photocatalytic efficiency of OG degradation compared to the pristine g-C_3_N_4_ (Figure 4b). This suggests that the active sites were not significantly affected by the polymer coating and interactions, and that there was no considerable reduction in the number of active sites upon encapsulation.

To compare the adsorption and photocatalytic behavior of the pristine g-C_3_N_4_ in different acidic conditions, the tests were also performed in neutral media (Figure 4b). The g-C_3_N_4_ showed negligible adsorption toward the OG dye in neutral media due to the presence of non-protonated electron-rich N-H groups in the g-C_3_N_4_ structure, which decreased the dye adsorption. This confirms the previous assumption that the protonated aliphatic nitrogen groups of g-C_3_N_4_ in neutral media were the most responsible for the better dye adsorption, leading to the conclusion that the OG dye interacted primarily with the g-C_3_N_4_ and not with the CMC/SA polymers. The degradation of the dye ceased after turning off the lamp, implying that both a catalyst and light irradiation are required for effective dye degradation (Figure 4b). The interaction between the dye and g-C_3_N_4_/CMC/SA composite hydrogels depends upon the structure and charge of the dye, and dictates whether the dye will interact with one composite component over another, with results emphasizing the critical role of the dye charge through favoring the adsorption or photocatalytic reaction.

The photocatalytic performance of the g-C_3_N_4_/CMC/SA_1:1_ and C_3_N_4_/CMC/SA_2:1_ beads have been compared and are presented in Figure 4c. The obtained results indicate that both composite hydrogels exhibited identical adsorption and catalytic performance, implying that different ratios of the CMC and SA polymers in the composite hydrogels do not exert any effect on the adsorption or photocatalytic performance. Several interesting observations could be drawn from this result: (i) both composite hydrogels possess the same number of available adsorption sites, being independent of the CMC/SA ratio in the polymer; (ii) nanoparticles have a similar distribution throughout both polymer matrices, and the obtained findings are in line with the theoretical assumptions, keeping in mind the similar chemical structures of the CMC and SA polymers and the presence of the same functional groups; (iii) the additional methylene group in the CMC polymer does not exert any effect on the adsorption nor the catalytic performance.

#### 2.2.2. Leaching and Stability Test

Good adhesion and high adhesion strength between the photocatalyst and the support are essential requirements for their practical application. Low adhesion on the support leads to the leaching of the active component of the catalyst from the support. One of the attractive methods to test the leaching of the g-C_3_N_4_ from the polymer matrix, and to indirectly verify the heterogeneous nature of the catalytic process, is to remove the catalyst from the reaction mixture before reaction completion, a protocol described by Sheldon et al. [55]. Thus, the composite hydrogel was removed from the reaction mixture after 1 h, when 40% of the OG dye was degraded and the reaction mixture was allowed to proceed further in the absence of the catalyst (Figure S2 in Supplementary Materials). If the leaching of the catalyst occurred, then the detached photocatalyst would form a fine suspension in the bulk solution, thus creating a pseudo-slurry effect. The results from our study revealed that the removal of the polymer decreased the reaction rate and no further noticeable degradation of the dye was detected after the continuation of the reaction time, proving the heterogeneous nature of the photocatalytic reaction and simultaneously substantiating the good adhesion of the g-C_3_N_4_ on the polymer matrix.

To evaluate the stability and long-term performance of the g-C_3_N_4_/CMC/SA beads, recyclability experiments were carried out. At the end of each cycle experiment, the photocatalyst was separated from the suspension, washed with water, and used in the next cycle with a fresh dye solution. Figure 4d shows the stability performance of the g-C_3_N_4_/CMC/SA_2:1_ beads over five cycles. The results demonstrated no significant changes in the photocatalytic activity up to five consecutive cycles; the degradation percentage of the OG dye declined from 98% in the first cycle to 87% in the fifth cycle. The recyclability test validated the good stability of the g-C_3_N_4_/CMC/SA_2:1_ beads under the applied cycling experiments, with a slight decrease in the decolorization percentage (a reduction rate of 13%). Moreover, the ATR-FTIR analysis was additionally conducted to evaluate the alterations in catalyst structure after the recycling experiments (Figure S3 in Supplementary Materials). The results revealed the nearly identical ATR-FTIR spectra of the g-C_3_N_4_/CMC/SA_2:1_ beads before and after the cycling tests. There was no appearance of new bands nor a change in the frequency and intensity of the existing characteristic C-N, C=O, or O-H bands compared to those in the initial g-C_3_N_4_/CMC/SA_2:1_ beads. Equally important, the obtained results indicated a good polymer stability, since light irradiation did not cause the photo-oxidation of certain side groups in the polymer chain.

#### 2.2.3. Radical Scavenger Study

To gain further insight into the photodegradation mechanism and to identify the major reactive species involved in the dye degradation, free radical scavenging experiments were performed by using IPA, EDTA, ASC, and AgNO_3_ as scavengers for ^•^OH, h^+^, O_2_^•−^, and e^−^, respectively. When a photocatalyst is irradiated by light, having an energy equal to or greater than the band gap, an e^−^ from the valence band is excited to the conduction band, leaving a positive h^+^ [56,57,58,59]. The photogenerated e^−^/h^+^ pairs can directly oxidize/reduce pollutant molecules or initiate a wide range of chemical reactions. The h^+^ can react with H_2_O or ^−^OH ions and oxidize them to ^•^OH, while e^−^ can react with O_2_ and reduce them to O_2_^•−^. The relevant reactions responsible for the photocatalytic degradation of the organic dye pollutant can be expressed as follows:

photocatalyst + *hν* → e_CB_^−^ + h_VB_^+^;
O_2_ + e^−^ → O_2_^•−^;
h^+^ + OG → intermediates and by-products;
O_2_ + 2e^−^ + 2H^+^ → H_2_O_2_;
H_2_O_2_ + e^−^ + H^+^ → ^•^OH + H_2_O;
h^+^ + OH^−^ → ^•^OH;
HO^•^/O_2_^•−^ + OG → intermediates and by-products.

**Figure 5:**
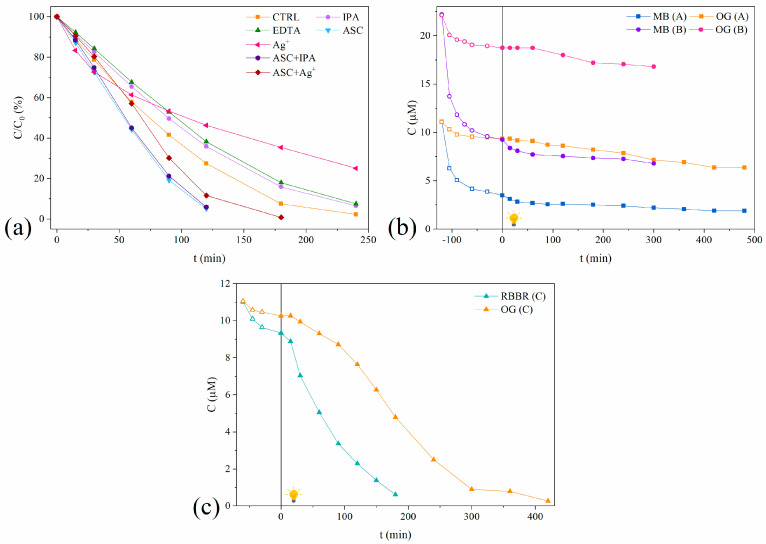
(**a**) Photodegradation activity of g-C_3_N_4_/CMC/SA_2:1_ beads in the presence of IPA, EDTA, ASC, Ag^+^, IPA/ASC, ASC/Ag^+^, and EDTA/ASC [c_0_ (OG) = 10 ppm, pH = 3.8, T = 25 °C, simulated solar light irradiation]; adsorption and photodegradation of (**b**) MB and OG and (**c**) RBBR and OG dyes in binary-dye solutions.

According to the reactions described above, the main reactive species are e^−^, h^+^, ^•^OH, and O_2_^•−^ radicals. Figure 5a presents the time-dependent photodegradation of the OG dye with selected radical scavengers under solar light irradiation using g-C_3_N_4_/CMC/SA_2:1_ beads. The zero reaction rate constants and half-degradation time of the control and radical scavenger reactions were determined and are presented in Table 5. The reduction in the reaction rate constants, denoted as *k*_scavenger_/*k*_control_, %, was used as a measure of an inhibitory effect in each reaction species.

The results showed that the addition of IPA had no effect, while the addition of EDTA slightly decreased the reaction rate, indicating that h^+^ had a greater impact on the OG photodegradation than ^•^OH. The addition of Ag^+^ into the reaction mixture triggered a remarkable decrease in the photocatalytic efficiency, suggesting a pivotal role of e^−^ in the degradation mechanism. Surprisingly, the addition of ASC as the O_2_^•−^ scavenger significantly enhanced the photodegradation efficiency of the OG dye. To the best of our knowledge, no studies have demonstrated an increase in the degradation efficiency with the addition of ASC.

From the literature findings, the contribution of each reactive species depends on the characteristics of the photocatalytic system, relying on both the structure of the pollutant and the type of the photocatalyst. Thus, it was found that both photogenerated O_2_^•−^ and h^+^ played a critical role in the Brilliant Green dye photodegradation using the 3D kaolinite/g-C_3_N_4_-alginate beads as a photocatalyst [24]. Additionally, O_2_^•−^ radicals are found to be the main reactive species in the photodegradation of MB using g-C_3_N_4_/diatomite/calcium alginate composite gel beads [60]. The g-C_3_N_4_-SA_15_ membrane was applied for Evans blue dye removal, and the use of sodium oxalate as an h^+^ scavenger, nitroxyl radical piperidinol as an O_2_^•−^, and IPA as the OH^•^ scavenger showed that the major active species were h^+^ and O_2_^•−^, with OH^•^ acting as a secondary active species [31]. The MB photodegradation using the ZnO/arabic gum photocatalyst showed a slight increase in the photodegradation rate in the presence of Ag^+^ and EDTA as radical scavengers of e^−^ and h^+^, respectively, concluding that both e^−^ and h^+^ take part in dye photodegradation [61].

The investigation presented in our study demonstrates that, depending on the reaction conditions, the ASC radical scavenger could play a role both as the O_2_^•−^ and h^+^ scavenger, thus serving both as oxidative and reductive agents. The results from our study distinguished electrons as the main reactive species. Therefore, the role of ASC is not only to scavenge O_2_^•−^, but also to act as an h^+^ sacrificial agent. By trapping h^+^, the ASC increased the number of electrons that could participate in the reaction, thus enhancing the degradation efficiency.

To further investigate the decisive role of ASC as a sacrificial agent, photodegradation experiments were conducted in the presence of two scavengers. In the first test, ASC served as an h^+^ scavenger and Ag^+^ served as an e^−^ scavenger. In the second test, ASC was used as an h^+^ scavenger and IPA was used as the ^•^OH radical scavenger. Compared to the control experiment, the presence of both ACS and Ag^+^ scavengers enhanced the photodegradation rate; however, the photodegradation rate was slower than when only ASC was used. This result could be attributed to e^−^ scavenging by Ag^+^ ions, leading to a reduced number of electrons in the reaction mixture. In contrast, the second test (with the ASC and IPA) showed an almost identical reaction rate constant, with a slightly increased *t*_1/2_, confirming that ^•^OH plays a negligible role in the degradation mechanism and is not formed through the h^+^-mediated process nor by the O_2_^•−^ process.

#### 2.2.4. Binary-Dye Photocatalytic Systems

To investigate the photocatalytic activity of g-C_3_N_4_/CMC/SA_2:1_ beads in binary-contaminant systems, and the mutual effect of dyes on their photoreactivity, representative solutions of MB, OG, and Remazol Brilliant Blue R (RBBR) dyes, as three contaminants of emerging concern, were employed. The first binary solution system consisted of the following two dyes: cationic MB and anionic OG dye (A and B solutions). Both solutions had the same MB to OG dye molar ratio but differed in their initial dye concentrations. The second binary-solution system (C) contained the following two anionic dyes: OG and RBBR dyes. The rationale behind mixing different dye types was to assess the potential synergistic effects on the photocatalytic degradation efficiency or the molecular repulsion between dye molecules, electrostatic interactions, or other types of intermolecular forces. Representative time-dependent kinetic curves for the photodegradation of MB and OG dyes in binary-dye solutions are presented in Figure 5b. Table 6 compares the adsorption capacity and photodegradation efficiency of the MB and OG dyes in single- and binary-dye systems.

The obtained results revealed the high adsorption of the MB dye onto the g-C_3_N_4_/CMC/SA_2:1_ beads in both single and binary MB-OG dye solution systems. The difference in the MB dye adsorption in single- and binary-dye solutions was a consequence of the different dye concentrations rather than the presence of another dye. On the other hand, there was a slight enhancement in the adsorption of the OG dye in the binary solutions compared to the adsorption in the single-dye solution. This is probably a result of an MB dye-promoting effect, in which the presence of adsorbed MB molecules on the composite surface may modify its surface properties, creating favorable adsorption sites or altering the surface chemistry in a way that enhances the OG dye adsorption. Additionally, the MB and OG dyes have complementary electrostatic properties, and the presence of positively charged MB molecules on the composite surface interacts favorably with negatively charged OG dye molecules, enhancing their adsorption through electrostatic attraction [50,51]. It is interesting that, in binary solutions, the adsorption of the OG dye remained consistent regardless of the concentration of MB dye. This suggests that the presence of MB dye, even at varying concentrations, did not significantly affect the adsorption behavior of the OG dye. According to the obtained results in the binary MB-OG dye solutions, MB dye photodegradation was firstly initiated, which was consistent with the detected higher adsorption onto the surface of the photocatalyst compared to the OG dye. Also, the results revealed that the degradation of the MB dye in the binary systems was faster than in the single system. This is likely due to different concentrations of MB dye rather than the effect of the OG dye, suggesting that the concentration of MB dye plays a dominant role in determining the degradation kinetics.

On the other hand, a significantly lower photodegradation rate of the OG dye was observed in the binary-dye systems compared to the single-dye system, which was influenced by several factors. The surface coverage by MB by-products partially blocks or passivates active sites, reducing the availability of the sites for OG dye adsorption and hindering its degradation. Additionally, the presence of MB degradation by-products indirectly affects the generation of reactive species involved in the photodegradation process. Interactions between MB degradation by-products and OG dye molecules may lead to the formation of complexes or aggregates with different photophysical or chemical properties, decreasing their susceptibility to photodegradation, and thus contributing to the lower degradation rate of the OG dye.

Two notable observations could be drawn from the obtained results. First, the degradation of the OG dye in solution A (with lower MB concentrations) followed one kinetic regime. In contrast, solution B (with higher MB concentrations) exhibited the following two successive kinetic regimes: the initial regime, with a very slow reaction rate, followed by the second regime, with a faster reaction rate. At the beginning of the reaction, the high MB concentration significantly influenced the OG dye adsorption and degradation. As the reaction proceeded, the concentration of MB dye decreased, and most of the catalyst surface became available, therefore facilitating the photodegradation of the OG dye. The transition from one kinetic regime to two kinetic regimes with the increasing MB concentration suggests a shift in the dominant factors influencing the OG degradation kinetics. In a single-kinetic regime, the degradation kinetics may be primarily governed by the availability of active sites on the photocatalyst surface. In a double-kinetic regime, the available adsorption sites on the photocatalyst may become saturated and the degradation kinetics could be influenced by additional factors. Another interesting conclusion is that the photodegradation rate constant, *k*, in solution A and the *k*_2_ of the second kinetic regime in solution B were similar despite the differences in the initial stages of degradation.

The second binary solution system studied consisted of the following two anionic dyes: OG and RBBR. In both the single and binary mixtures, the molar ratio of RBBR to OG remained the same, but the initial concentration of each dye was twice as high in the single mixture compared to the binary mixture. Representative time-dependent kinetic curves for the photodegradation of OG and RBBR in single- and binary-dye systems are presented in Figure 5c and Figure S4 in Supplementary Materials. Table 6 compares the adsorption capacity and photodegradation efficiency of the RRBR and OG dye in single- and binary-dye systems. Both dyes exhibited poor adsorption onto the hydrogel composite, whereas the RBBR showed higher adsorption affinity than the OG. The difference in the adsorption affinity could be attributed to various factors, including the dye molecular structure, the nature of the functional groups, steric effects, or interactions with the surface of the beads. RBBR, an anthraquinone dye, consists of three fused rings with carbonyl (C=O) groups and three sulfonic groups (-SO_3_H), as well as NH_2_ and NH groups. In contrast, OG dye features two aromatic rings linked by one azo group (-N=N-). The adsorption of both dyes was slightly reduced in the binary solution compared to the single-dye solution, implying that the presence of another dye and any potential interactions between the dyes might not significantly affect the adsorption behavior in the binary system.

In single-dye solutions, the photocatalytic degradation efficiency of both the RBBR and OG dyes was similar. Despite RBBR showing a higher affinity for the catalyst surface compared to OG in single-dye solutions, the photocatalytic degradation efficiency of both dyes remained similar. In binary solutions, a significant reduction in the photodegradation efficiency was obtained, which was more pronounced for the OG dye compared to the RBBR dye, suggesting that the presence of another dye in the solution affects the photocatalytic degradation process. The RBBR dye exerted a stronger influence on the degradation of the OG dye in binary solutions compared to the reverse set-up.

Furthermore, the degradation of the RBBR dye can be described with one kinetic regime, while the degradation of the OG dye follows two successive kinetic regimes, further supporting the inhibitory effect of the RBBR dye on the degradation of the OG dye. In the binary-dye solution, the number of active sites reduced as they were occupied by both dyes, resulting in a decreased degradation efficiency. The adsorption percentage of the RRBR dye was two times higher compared to the adsorption percentage of the OG dye, indicating that there were twice as many available active sites for the RBBR dye. This resulted in the apparent reaction rate constant being halved for the RBBR dye and reduced by three times for the OG dye. The structural disparities between the RBBR and OG dyes may be a reason for the variations in their reactivity and the formation of different degradation pathways during photocatalytic processes. The photodegradation of the RBBR dye started with the removal of amino and sulfonic groups from the dye structure, followed by the degradation of the quinone structure and sulfonic acid derivatives into simple organic compounds [62]. On the other hand, it could be suggested that the photodegradation of the OG dye began with the electrophilic attack on the azo group. In the low-pH solution, the excess H^+^ concentration led to the interaction between the H^+^ and azo linkage, consequently decreasing the electron density of the azo group and initiating a higher resistance to radical attacks [51].

## 3. Materials and Methods

### 3.1. Synthesis of Composite Hydrogel

The synthesis of the g-C_3_N_4_ was conducted using a facile thermal polymerization of precursors. Briefly, 2.0 g of urea (Sigma-Aldrich, St. Louis, MO, USA) and 2.0 g of melamine (Sigma-Aldrich, St. Louis, MO, USA) were added into a ceramic crucible, making a physical mixture, and left in an annealing oven at 550 °C for two hours (step ramping rate of 10 °C min^−1^). After cooling down to room temperature, the g-C_3_N_4_ was put into a 20% ethanol solution for a few hours in order to achieve ultrasonic dispersion on an ultrasonic device and dried at 80 °C afterward.

**Figure 6:**
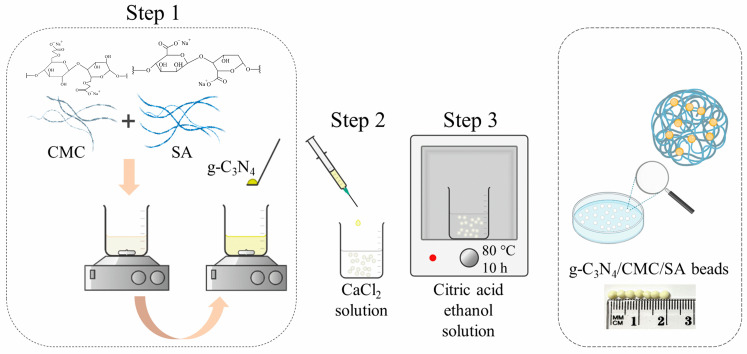
Experimental set-up of composite hydrogel synthesis.

For better comprehension, the overall step-by-step experimental set-up of the composite hydrogel synthesis is presented in Figure 6. Polymer solutions with different CMC/SA ratios (2:1 and 1:1) were prepared by dissolving 375 mg of the total amount of polymers, CMC (Tokyo Chemical Industry, Tokyo, Japan) and SA (Sigma-Aldrich, St. Louis, MO, USA), in 15 mL of distilled water with continuous stirring. Simultaneously, 75 mg of g-C_3_N_4_ was added to each polymer solution until complete solubilization was reached. Thereafter, using a plastic syringe and syringe pump (Aladdin 1000 Programmable Syringe Pump, World Precision Instruments, Sarasota, FL, USA) under gentle stirring, the g-C_3_N_4_/CMC/SA composite mixture was added into the CaCl_2_ solution (2.5%) as a gelling medium. The third step was the addition of the g-C_3_N_4_/CMC/SA composite hydrogel beads into a 2.9 wt. % citric acid (CA) ethanol solution for additional crosslinking, which was aged for 10 h at 80 °C. The prepared g-C_3_N_4_/CMC/SA beads were washed with ethanol and distilled water, and stored in distilled water in a refrigerator at 3 °C. The detailed composition of the synthesized polymer and composite hydrogels and sample codes are presented in Table 7. For sample characterization, the polymer and composite hydrogels were additionally dried by freeze-drying.

### 3.2. Characterization of Composite Hydrogel

A field-emission scanning electron microscope (Tescan FE-SEM Mira 3 XMU, Tescan a. s., Brno, Czech Republic) operated at 20 keV was used to characterize the morphology of the samples. The EDX spectra were recorded with an Oxford Inca 3.2 energy-dispersive spectroscopy ( Oxford Instruments, Oxford, UK) coupled with a Jeol JSM 5800 SEM (Jeol Ltd., Tokyo, Japan) to analyze the presence of elements on the surface of the samples. The XRD analysis was carried out using the Rigaku Smart Lab instrument (Rigaku, Tokyo, Japan) under Cu-Ka 1.2 radiation. The diffraction pattern was measured with a continuous angular scanning of 2° min^−1^ collected at 0.02° intervals. The chemical structures of the hydrogels were characterized by ATR-FTIR spectroscopy (Thermo Fisher Scientific, Waltham, MA, USA) in the range of 400–4000 cm^−1^ with a 2 cm^−1^ scanning resolution in the temperature range between 25 and 1200 °C under an airflow. The TGA was performed by a TA SDT 2960 instrument (TA Instruments, New Castle, DE, USA) under a nominal argon flow of 30 mL min^−1^ in the temperature range of 25–800 °C and at a heating rate of 10 °C min^−1^. The single compression tests were performed at room temperature using a Shimadzu Autograph AGS-X (1 kN) testing machine (Shimadzu, Kyoto, Japan) at a constant strain rate of 2 mm per minute. The hydrogels were compressed to the moment of the network collapse of the hydrogels. The test results are given as an average value from three independent measurements. The DRS measurements of samples were performed using a Shimadzu UV–visible UV-2600 spectrophotometer (Shimadzu, Kyoto, Japan) equipped with an integrated sphere ISR-2600 Plus. The obtained data were processed by Kubelka–Munk transformation. The pore size, pore volume distribution, and porosity were determined by MIP on a Pascal 140/440, Thermo Scientific (Thermo Fisher Scientific, Waltham, MA, USA).

### 3.3. Photocatalytic Activities and Radical Scavenger Study

The photocatalytic activity of the obtained g-C_3_N_4_/CMC/SA_2:1_ beads was tested using model pollutants, OG, MB, and RBBR dyes, in aqueous solutions. All photocatalytic experiments were performed in an open cylindrical thermostatic Pyrex cell photocatalytic reactor using an Osram Ultra Vitalux 300 W lamp, whose spectrum simulates sunlight, positioned 50 cm above the surface of the reaction solution. The reaction vessel temperature was maintained at 25 °C using a thermostat Julabo F25 (Julabo Labortechnik GmbH, Seelbach, Germany), and stirring of the reaction suspension was accomplished on a magnetic stirrer. The changes in the dye concentration in the solution were monitored by a UV–Vis spectrophotometer (Thermo Electron Nicolet Evolution 500, Thermo Fisher Scientific, Waltham, MA, USA) by recording the absorption spectrum of the dye solution. Prior to each photocatalytic analysis, 6.8 g of the g-C_3_N_4_/CMC/SA (400 beads with 75 mg g-C_3_N_4_) was added into the reactor together with the dye solution and stirred in the dark until adsorption/desorption equilibrium between the dye and the catalyst surface was reached. After the equilibrium was achieved, reaction mixtures were then irradiated and aliquots were taken and analyzed at predetermined time intervals, measuring the absorbance spectrum of the dye solution.

The photodegradation of the OG, MB, and RBBR dyes was tested in single- and binary-dye systems. To examine the binary mixtures, two MB-OG binary-dye solutions were prepared with the same MB to OG dye molar ratio (50:50) but different initial dye concentrations. The second binary-solution system contained OG and RBBR dye in a 50:50 molar dye ratio with only one initial dye concentration.

## 4. Conclusions

In this study, environmentally friendly g-C_3_N_4_/CMC/SA composite hydrogels were successfully developed from eco-friendly precursors through a simple two-step crosslinking process. The g-C_3_N_4_ nanoparticles were uniformly dispersed into the CMC/SA matrix, resulting in a homogeneous, highly porous composite structure with enhanced thermal stability and prolonged elongation. The structure and charge of the dye molecules were crucial in determining their interactions with the composite constituents, the g-C_3_N_4_ and CMC/SA copolymers. The results indicate that the OG and RBBR dyes predominantly interacted with the g-C_3_N_4_ rather than the CMC/SA copolymers, which was contrary to the MB dye, which predominantly interacted with the CMC/SA copolymers. The synthesized composites exhibited a high adsorption capacity and a low photocatalytic efficiency toward the cationic MB dye. Conversely, the synthesized composites showed a low adsorption capacity and a high photocatalytic efficiency towards the anionic OG dye. This outcome was due to the weaker interactions between the negatively charged OG dye molecules and the composite material’s surface. Furthermore, the composite retained photoefficiency after multiple cycles, indicating not only the long-term performance of the composite and the good adhesion of the g-C_3_N_4_ on the polymer matrix but also the high photostability of the polymer matrix. The results from the scavenger study revealed that photo-induced electrons are the primary reactive species responsible for the photodegradation of OG dye under solar light irradiation. In the cationic/anionic binary-dye systems, the photodegradation efficiency of the OG dye was notably affected by the presence of the MB dye, resulting in its reduced susceptibility to degradation. In the anionic/anionic binary-dye system, the RBBR dye exerted a stronger influence on the degradation of the OG dye compared to the reverse set-up. The results from the present study suggest that utilizing eco-friendly g-C_3_N_4_/CMC/SA composite hydrogel, with a high adsorption capacity coupled with excellent photoefficiency and photostability, good mechanical properties, and high thermal stability, offers a sustainable and efficient solution for environmental remediation, particularly in the removal of organic pollutants. Furthermore, its reusability and the absence of the need for additional separation processes enhance its overall sustainability, making it a valuable tool in the quest for cleaner and healthier environments.

## Figures and Tables

**Table 1:** Temperature values at 5% and 30% weight loss and the heat-resistance index of the CMC/SA_1:1_, CMC/SA_2:1_, g-C_3_N_4_/CMC/SA_1:1_, and g-C_3_N_4_/CMC/SA_2:1_ beads.

Sample	T_5%_, °C	T_30%_, °C	T_HRI_, °C
CMC/SA_1:1_	168	259	109
CMC/SA_2:1_	159	248	104
g-C_3_N_4_/CMC/SA_1:1_	169	287	117
g-C_3_N_4_/CMC/SA_2:1_	178	301	123

**Table 2:** Mechanical properties of the hydrogels.

Sample	^a^ σ, N/mm^2^	^b^ MSS, %
CMC/SA_1:1_	0.1548	59.94
CMC/SA_2:1_	0.1735	39.48
g-C_3_N_4_/CMC/SA_1:1_	0.1539	62.91
g-C_3_N_4_/CMC/SA_2:1_	0.0988	47.98

^a^ maximal compressive stress; ^b^ maximal stroke strain.

**Table 3:** MIP measurements of the pristine polymer hydrogels and composite hydrogels.

Sample	^a^ V_p_ (mm^3^ g^−1^)	^b^ D_p_ (µm)
CMC/SA_1:1_	7489	23
CMC/SA_2:1_	2036	23
g-C_3_N_4_/CMC/SA_1:1_	7181	4
g-C_3_N_4_/CMC/SA_2:1_	5663	24

^a^ total intruded mercury volume; ^b^ median pore diameter.

**Table 4:** Comparison of g-C_3_N_4_/CMC/SA_2:1_ photocatalytic activity with the other published results.

Photocatalyst Material	Pollutant	Pollutant Concentration, ppm	Removal Efficiency	Ref.
Ag/AgCl/Al-CMC	Rhodamine B	10	98% in 60 min	[52]
Ag/AgCl/Fe-CMC	Rhodamine B	10	87% in 60 min	[52]
CMC/polyanilyne/TiO_2_	Cristal Violet	50	99% in 80 min	[53]
g-C_3_N_4_/calcium alginate microsphere	Rhodamine B	5	97% in 34 h	[39]
g-C_3_N_4_/calcium alginate microsphere	MB	10	81% in 42 h	[39]
g-C_3_N_4_/calcium alginate beads	Rhodamine B	10	98% in 60 min	[40]
CuO/g-C_3_N_4_/calcium alginate	Tetracycline	13	75% in 60 min	[41]
nitrogen-doped graphene oxide/ZnO/ZnO_2_/CMC	Methyl Orange	10	99% in 200 min	[54]
nitrogen-doped graphene oxide/ZnO/ZnO_2_/CMC	Rhodamine B	50	99% in 160 min	[54]
g-C_3_N_4_/CMC/SA_2:1_	OG	10	98% in 180 min	Present work

**Table 5:** Photocatalytic degradation of OG dye using g-C_3_N_4_/CMC/SA_2:1_ beads with added scavengers.

Scavengers	Limiting Species	^a^ *k* [μmol dm^−3^ min^−1^] × 10^2^	^b^ *k*_scavenger_/*k*_control_, %	^c^ *t*_1/2_, min
CONTROL	/	12.0	/	82
IPA	^•^OH	11.8	98.3	89
EDTA	h^+^	10.9	90.8	95
ASC	O_2_^•−^/h^+^	18.6	155	55
Ag^+^	e^−^	6.6	55.0	129
IPA/ASC	^•^OH and O_2_^•−^/h^+^	18.3	152	56
ASC/Ag^+^	O_2_^•−^/h^+^ and e^−^	15.4	128	64

^a^ photocatalytic rate constant; ^b^ the ratio between the radical scavenger-inhibited reaction rate and control constants; ^c^ half-life time of the photocatalytic reactions.

**Table 6:** Adsorption and photodegradation efficiency of MB, OG, and RBBR dyes in single- and binary-dye solutions.

Solution	Dye	Total Initial Dye Concentration, μmol dm^−3^	Dye Concentration, μmol dm^−3^	^a^ *R*, %	^b^ *q*_e_, mg g^−1^	^c^ *k* [μmol dm^−3^ min^−1^] × 10^2^
Binary solutions, MB-OG						
A	MB	22.2	11.1	68.6	0.042	1.3
	OG		11.1	15.3	0.011	0.73
B	MB	44.2	22.1	58.4	0.071	3.8
	OG		22.1	15.2	0.022	*k*_1_ = 0.014; *k*_2_ = 0.8
Binary solutions, RBBR-OG						
C	RBBR	22.2	11.1	15	0.015	7.0
	OG		11.1	7	0.0053	*k*_1_ = 2.0; *k*_2_ = 4.0
Single solutions						
	MB	13.3	13.3	64	0.031	2.0
	OG	22.1	22.1	11	0.016	12.0
	RBBR	22.1	22.1	21	0.042	14.0

^a^ adsorption percentage of dye; ^b^ adsorption capacity (mg dye per g of the photocatalyst); ^c^ photocatalytic degradation rate constant.

**Table 7:** Composition of synthesized polymer and composite hydrogels and sample codes.

Sample Codes	CMC Content, mg	SA Content, mg	g-C_3_N_4_ Content, mg
CMC/SA_1:1_	187.5	187.5	/
CMC/SA_2:1_	250	125	/
g-C_3_N_4_/CMC/SA_1:1_	187.5	187.5	75
g-C_3_N_4_/CMC/SA_2:1_	250	125	75

## Data Availability

Data is contained within the article and Supplementary Materials.

## Supplementary Materials

The following supporting information can be downloaded at: https://www.mdpi.com/article/10.3390/ijms25147896/s1.
