# Supplementary material for: Eco-Friendly g-C3N4/Carboxymethyl Cellulose/Alginate Composite Hydrogels for Simultaneous Photocatalytic Degradation of Organic Dye Pollutants
Source: Int J Mol Sci. 2024 Jul 19;25(14):7896. doi: 10.3390/ijms25147896 (PMC11277058; doi:10.3390/ijms25147896)
Supplement: Supplementary file 1 [file ijms-25-07896-s001.zip › ijms-3089972-supplementary.pdf]

Supplementary Materials

Eco-Friendly g-C_3N_4/carboxymethyl cellulose/alginate Composite Hydrogels for Simultaneous Photocatalytic Degradation of Organic Dye Pollutants

Ksenija Milošević[1], Davor Lončarević[1], Melina Kalagasidis Krušić[2], Milica Hadnađev-Kostić[3], Jasmina Dostanić[1]

[1] Department of Catalysis and Chemical Engineering, Institute of Chemistry, Technology and Metallurgy, National Institute of Republic of Serbia, University of Belgrade, Njegoševa 12, 11000 Belgrade, Serbia

[2] Faculty of Technology and Metallurgy, University of Belgrade, Karnegijeva 4, 11000 Belgrade, Serbia

[3] Faculty of Technology Novi Sad, University of Novi Sad, Bulevar Cara Lazara 1, 21102 Novi Sad, Serbia

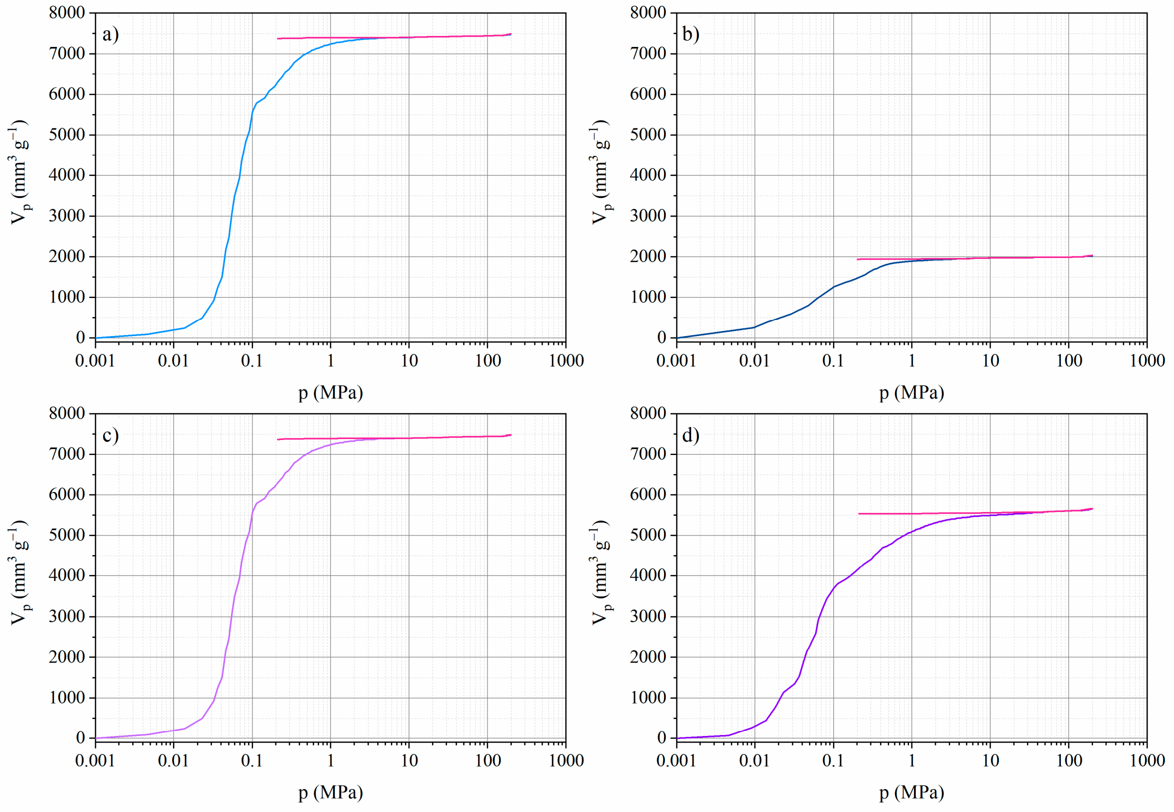

Figure S1. Intrusion and extrusion curves for mercury porosimetry measurements of a) CMC/$SA_{1:1}$, b) CMC/$SA_{2:1}$, c) g-C_3N_4/CMC/$SA_{1:1}$, and d) g-C_3N_4/CMC/$SA_{2:1}$ samples.

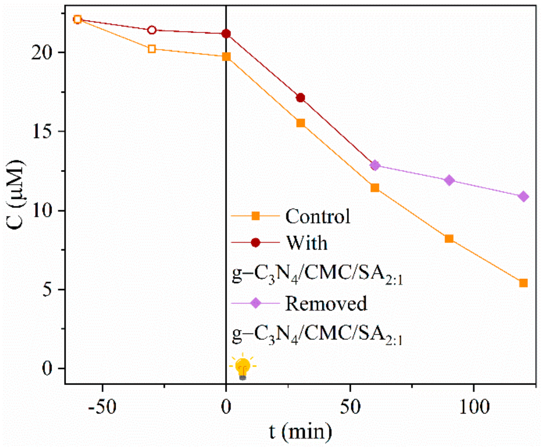

Figure S2. Photodegradation of OG dye using g-C_3N_4/CMC/$SA_{2:1}$ beads. [Experimental conditions: c0 (OG)=10 ppm, pH=3.8, T=25 °C, simulated solar light irradiation]

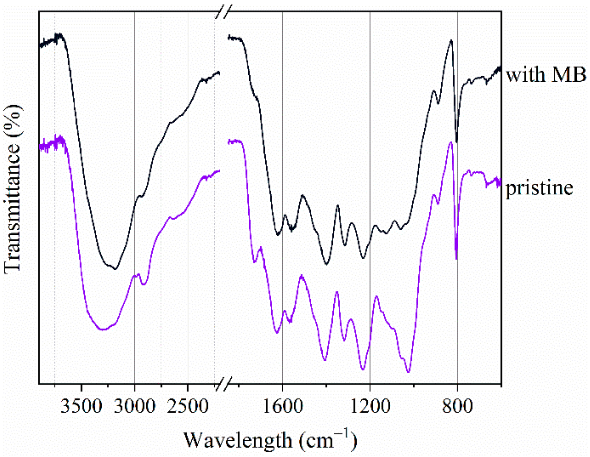

Figure S3. ATR-FTIR spectra of pristine nanocomposite hydrogels and nanocomposite hydrogels after MB dye photodegradation.

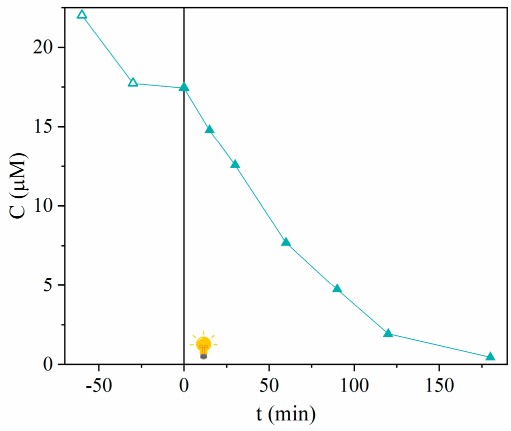

Figure S4. Photodegradation of RBBR dye using g-C_3N_4/CMC/$SA_{2:1}$ beads. [Experimental conditions: c_0 (RBBR)=13.8 ppm, pH=3.8, T=25 °C, simulated solar light irradiation]
